# Tools for the Approach of Fear, Anxiety, and Stress in the Domestic Feline: An Update

**DOI:** 10.1155/vmi/9109397

**Published:** 2025-08-06

**Authors:** Florencia Barrios, Paul Ruiz, Juan Pablo Damián

**Affiliations:** ^1^Departamento de Clínicas y Hospital Veterinario, Facultad de Veterinaria, Universidad de la República, Montevideo 13000, Uruguay; ^2^Núcleo de Bienestar Animal, Facultad de Veterinaria, Universidad de la República, Montevideo 13000, Uruguay; ^3^Departamento de Biociencias Veterinarias, Facultad de Veterinaria, Universidad de la República, Montevideo 13000, Uruguay

## Abstract

Domestic cats are one of the most popular pets worldwide. Despite their growing popularity, there remains a significant gap in understanding their specific needs, leading to frequent challenges in human–cat coexistence and behavioral issues. The distinct emotional systems of cats, compared to other domestic species and humans, complicate the interpretation of feline behavior. Notably, fear, anxiety, and stress are among the most prevalent behavioral problems in cats. This review delves into the core concepts of anxiety, fear, and stress responses in domestic cats, highlighting their unique characteristics and providing recommendations for effective handling. Additionally, it presents some key international recommendations for creating a “cat-friendly” environment, as well as suggestions for pharmacological management to ensure a stress-free visit to the clinic for the cat, its caretaker, and the veterinary team.

## 1. Introduction

Currently, the domestic cat (*Felis silvestris catus*) is one of the most popular companion animals in the world [1, 2]. However, despite their great popularity, there is a significant lack of understanding of their species-specific needs. Archaeological data show that domestic felines have lived with humans for roughly 9500 years [3], but dogs have records dating back more than 15,000 years [4]. Lately, available genetic tools have brought us closer to domestic felines, revealing that their closest ancestor is the African wild cat (*Felis silvestris lybica*) [5]. This wild feline still exists today and is considered as Least Concern in the IUCN Red List of Threatened Species [6]. They currently reside across North Africa, sub-Saharan Africa, the Arabian Peninsula, and parts of Western Asia, including regions like Israel, Syria, Iraq, and western Iran [6]. They are mostly solitary, do not live in groups, and do not tolerate the presence of other members of their own species, with the exception of the breeding season, where copulation and rearing of kittens by the female occur [7].

A curious fact is that the domestic cat is not even considered a completely domesticated species by many authors who prefer the term “tamed” [1]. The term began to be used after observing the great ease with which it transitions from being a feral individual to a tamed one in a few generations [8]. Also, considering that it does not exhibit all the characteristics of a domesticated species itself, such as selective reproduction, dependence on humans for survival in a wild environment, and significant morphological changes from their wild ancestors, among others [8]. As expected for a species that is inherently solitary, 9500 years of coexistence with other cats have not been enough for developing typical social communication tools like other species. Since cats lack passive conflict-resolution strategies and reconciliation behavioral tools, they often struggle to resolve conflicts and promote group cohesion [9, 10], often leading to tension and confrontation. This is one of the reasons why harmonious coexistence is generally difficult in multicat households without proper handling and environmental measures.

Undoubtedly, the remaining characteristic traits of *F. silvestris lybica* existing within domestic felines have generated vast difficulties regarding adaptation and coexistence with other cats and humans, especially when our expectations are often related to our experience with the canine species. A significant portion of the behavioral problems exhibited by cats are related to stress and anxiety triggered by human impositions that threaten the cat's normal behavior, such as forced group coexistence, territory restriction, scarce distribution, and number of resources, among others [1, 11]. On the other hand, the cat has acquired some advantages from domestication since, through humans, it has managed to turn from an animal that's practically intolerant of others of the same species into “facultative social” individuals that coexist in groups with other cats if appropriate conditions are provided [12–15]. Because cats were domesticated relatively recently compared to other species, many of their habits resemble those of their ancestors [16]. This is demonstrated by their preference for hunting, playing, exploring, territorial marking, and distinct reproductive cycles [1, 2, 16, 17]. Some of these traits may contribute to typical behavioral concerns, including fear and anxiety, which are frequently misunderstood or missed by their human caretakers.

We share many ties with cats, but like any domestic species (or those in the process of becoming one), humans have sought ways to use them for different purposes apart from being a pet. Cats started as pest controllers [18], were then fur animals [19], and closer to the last century, became a pet and also a model of neurobiology [16]. Because of the significant similarity between the nervous systems of cats and humans, cats have long been considered the most suitable model for comparative studies in neurology and anatomy [20–23]. However, their use has substantially decreased in recent years, possibly because of pressure from social groups [24].

## 2. Neurobiology of Cats, Emotions, and Feelings

Cats and humans share lobes and a cerebral cortex with similar structures, although we have several anatomical differences [25]. Cerebral cortex areas that exhibit similarities in cytoarchitectonic organization appear to be connected in an analogous manner [26]. Their central nervous system (CNS) organization, particularly in the cerebral cortex and spinal regions, is a useful model for human brain research. Lately, cats were mostly being used as models for researching neurological illnesses, similar to those found in humans [21, 27]. Cats' motor cortex arrangement is very similar to ours, allowing researchers to draw parallels between motor function and neurological disease outcomes [28]. Cats are used to study conditions such as multiple sclerosis [29] and epilepsy [30] because of their similar sensory integration and pathway disorders. This similarity facilitates the development of treatment methods and a better understanding of neurodegenerative processes.

Like other mammals, the primary structures of the nervous system involved in fear, anxiety, and stress in domestic felines are comparable (thalamic tracts, amygdala, and hypothalamus), indicating a close evolutionary relationship to a certain extent [31]. The hypothalamic–pituitary–adrenal (HPA) axis in cats has been investigated alongside the human stress response systems. Insights gained from researching how animals react to fear, anxiety, or stress can be transferred to human reactions [32–34], resulting in a more complete knowledge of stress-related diseases such as PTSD and persistent anxiety. Neuroanatomical studies in cats help to explain the pathways and interconnections within the HPA axis, providing prospects for creating targeted treatments that modify this system in humans. Furthermore, compulsive illnesses in cats frequently mirror human obsessive–compulsive disorder (OCD) due to aberrant serotonergic transmission, as demonstrated by their reaction to serotonin reuptake inhibitors [35, 36].

Despite the extensive knowledge gained about their neuroanatomy, this was not the same for emotions [37]. This delay has coincided with a reluctance to consider emotional health as a crucial aspect of veterinary medicine [38]. To understand the important link between emotional and physical welfare, we need to understand how cats feel and emotionally perceive the world around them. We could start by distinguishing between emotions and feelings. Feelings are the subjective and conscious experiences of emotions [39] as a result of the need to understand and evaluate the observed emotional state at a given moment. Discussions regarding feelings in nonhuman animals are problematic since they frequently include a more introspective understanding of experienced emotions. It is important to explore the relationship between feelings, fear, and anxiety as sources of stress, with fear acting as a psychological stressor.

Emotions are complex reaction patterns that include sensory, behavioral, and physiological characteristics as an individual tackles a personally relevant subject or event [40]. These emotions include fear and anxiety. Another definition describes emotions as a broad notion that encompasses emotional, cognitive, behavioral, expressive, and a variety of physiological changes inside an individual [41]. This distinction is very important in veterinary practice, as understanding and resolving cats' emotional states can have a significant impact on their treatment and overall welfare. In Feline behavioral medicine, the term “emotion” refers to motivational–emotional processes that cause “instinctive emotional arousal” in cats [42]. This refers to cats' natural (often instantaneous) physiological response to certain triggers, which might range from loud noises to the presence of unknown humans or animals. This response derives from the cat's core survival instincts, which include the fight-or-flight response. When a cat is excited, it may hiss, blow up its fur, or escape the scene. These reflexes are triggered by the cat's natural feelings and aim to defend them from perceived threats. In most animals, including cats, we can describe emotions as positive or negative [42, 43], with multiple emotional systems identified [41] as described in Table 1.

Also, feline emotions are perceived and classified based on not just their valence (positive or negative), but also their level of excitement (“arousal”). Heath [38] defines this term as the “amount of emotion that the animal presents at a given moment, regardless of valence.” Understanding these emotional systems allows veterinarians to tailor their approaches to feline patients, resulting in more effective and compassionate care.

## 3. Ontogeny of Fear, Anxiety, and Stress in Cats

Kittens are born altricial, so at first they are heavily reliant on their mother. For their first days, their eyes and ears are closed (they are blind and deaf) and they have limited movement and little hair. Their eyes typically open 7–14 days after birth, but complete vision and depth perception take longer to develop, maturing around Week 8 [18]. Hearing develops in the second week, and by the fourth week, kittens can hear and respond to auditory signals [44]. Understanding cat behavior involves a lot of concepts, including kittens' sensory development [45], mother and siblings' effect, genetics, past experience, and temperament, among others. The queen (mother cat) is vital in helping kittens overcome fear and develop coping abilities. Kittens rely on their mothers not only for food but also for guidance in understanding their environment, the mother's responses to stimuli influence the kittens' reactions. Fear responses can start to develop as early as 2–3 weeks of age, when kittens start to interact with and perceive their surroundings. A key period for socialization spans between the ages of 2 and 7 weeks, when kittens are most responsive to external stimuli and learning does not elicit a fear response [45]. Beyond this time frame, unknown objects, people, or events can cause fear and anxiety, indicating that the primary socialization window has closed [46]. It is particularly difficult for cats to form bonds with humans if they have not been adequately socialized at the appropriate developmental window [18] or if the mother perceives humans as a threat [16].

As in other mammals, maternal stress can negatively impact kittens, potentially resulting in increased fear or anxiety in litters [47]. McCune [48] found that the mother's disposition can influence kittens' stress levels and exploratory behaviors. According to other studies, kittens reared by highly reactive mothers exhibit heightened fear responses and a higher delay when approaching novel items [49, 50]. Siblings also make a major contribution to behavioral development through social play, which peaks between 9 and 16 weeks. This social play encourages the development of social skills, aggression control, and physical coordination. Kittens removed too early from their littermates or without siblings usually exhibit social behavior deficits and inappropriate aggression or fear responses toward other cats or people [51], especially before 9 weeks [52]. These kittens may show greater reactivity in stressful situations [53, 54], aggression [55], anxiety-related disorders as adults [50], and stereotyped behaviors [55, 56]. The above, far from being a list of problems associated with the kitten's bond with their mother and littermates, intends to highlight the importance of considering the cat as more than its own personality or genetics, but as an individual shaped by many aspects combined. This holistic vision of the cat not only improves research, but also breeding practices through devising tools for early intervention and socialization strategies. Also, it allows for practicing comprehensive and evidence-based medicine since welfare is a triad of cognitive, physical, and emotional health [38].

### 3.1. Fear in Cats

Fear is an emotional state of heightened arousal that happens when an animal meets a particularly risky situation, which usually results in defensive or escape reactions [57]. It is an unpleasant but natural physiological state that cats need for survival [58], but it is also a powerful stressor that causes anxiety and may lead to avoidance or aggression. If fear is thought to be the root cause of a patient's condition, an accurate environmental, proximal, and distant history must be gathered. The accuracy of fear classification is crucial for treatment effectiveness and the selection of appropriate therapies, such as medication, environmental enrichment, habituation, or others [59]. In veterinary behavioral medicine, three degrees of fear are typically acknowledged [59], simple fears, complex fears, and phobias.

Simple fears can be defined by their distinct response to specific stimuli, such as avoidance or exploration behavior following exposure. This category includes stimulus like unexpected loud noises or interaction with unknown people. On the other hand, when the fear response involves other factors called complex fear. These are possibly associated with increased anticipation and anxiety, as well as reactivity to a wide range of events, and are linked to experiences or stimuli that are generally already known to the cat. Complex fears include meeting with veterinarians or being in strange surroundings.

Finally, phobias are described as a state of very intense fear that is disproportionate to the triggering stimulus. These responses rarely contain avoidance behaviors and may consist of a brief or irrelevant reaction followed by a flight or escape behavior. Phobias are linked to a “panic” response [59], like fear of water or fear of confinement. The term “phobia” is rarely used to describe feline behavior, although it is used more frequently in canine ethology.

Fear is a complex response linked to one or more concomitant stimuli, so it should ideally be addressed in a multidisciplinary manner and through specific approaches such as desensitization techniques. One interesting desensitization approach for this is positive reinforcement training, which uses highly palatable food in the presence of the stimulus or in the location where the fear response is elicited [17]. Always consider that for most cats (50%), social interaction with humans is even more rewarding than the food itself which contradicts popular belief [60]. This allows professionals to work effectively with cats who cannot consume palatable treats (for example, for dietary or health reasons) or that are not food-motivated, and still be able to work successfully through these therapeutic approaches.

### 3.2. Anxiety in Cats

Anxiety is the emotional anticipation of a future event that causes cognitive, behavioral, and affective changes due to uncertainty about possible dangers [61]. A variety of factors can influence anxiety development in cats, including genetics, early life experiences, and environmental conditions. Environmental factors, such as loud noises or changes in the home, might worsen cats' anxiety, emphasizing the need for a stable and enriching environment [62].

Among the most common signs of anxiety in animals are hypervigilance (most common in cats), prolonged wakefulness, vocalizations, yawning, lip licking, tremors, hypersalivation, panting, urination/defecation, vomiting, diarrhea, decreased appetite, hiding, and escape attempts [63]. Chronic anxiety and related stress affect homeostasis and may potentially lead to diseases such as “compulsive disorders.” OCD is defined as repeating ritualistic actions that go beyond what is required for normal functioning, interfering with everyday tasks, and overall functioning [64]. In humans, OCD is a widespread mental health disorder characterized by repeating unpleasant thoughts, desires, or visions (obsessions), as well as repetitive behaviors or mental acts (compulsions) that patients feel compelled to perform in response to obsessions or strictly enforced rules. This disorder has a major impact on quality of life [40]. According to research, there is a genetic and environmental predisposition to develop OCD in people, including persistent stress and anxiety. As far as we know, OCD affects humans, dogs, and cats, who exhibit specific condition-related behaviors. Cats exhibit an OCD-like behavior called psychogenic alopecia (or excessive grooming). Cats with this disorder excessively groom their fur, resulting in hair loss and in severe cases, skin lesions. This behavior is thought to be stress-related and is frequently induced by environmental changes or stressors [65].

Prolonged stress in animal species also leads to mental health-related diseases, particularly those involving obsessive behavior. Fear and anxiety are the most common behavioral disorders seen in small animal practice [35], and therefore, its impact should not be overlooked. Treatment for these varies depending on the species and individual needs, but typically includes a combination of behavioral modification strategies, environmental enrichment, and, in some circumstances, pharmacological treatment.

### 3.3. Stress in Cats

Stress is described as a homeostatic imbalance [40] in response to external or internal variables that causes changes, which may influence how an individual feels and behaves [40]. The stress response is coordinated by two primary systems: the sympatho-adrenomedullary axis and the hypothalamic–hypophyseal–adrenal (HHA) axis [66–69]. When the HHA axis is activated, corticotropin-releasing factor (CRH) enters the bloodstream, increasing the production of adrenocorticotropic hormone (ACTH), thereby increasing serum cortisol [70]. This efficient system normally has negative feedback and is very efficient for the survival of the individual through short-term response of fight or flight (or freeze) behavior. When normal, it is called “eustress,” and in a short period of time, it allows to safeguard the safety of the individual, after which vital signs quickly return to baseline values. Stress should not be regarded as pathologic at first because it is a normal physiological reaction that all animals experience to varying degrees [71]. Stress reactions develop from infancy to adulthood and are influenced by genetic, environmental, and social variables. Variations in genes associated with neurotransmitter systems such as serotonin and dopamine can alter an animal's susceptibility to anxiety and stress, just as they do with humans. Overall, [72] suggests that differences in these genes may explain why some cats are more resilient, whereas others are more sensitive to changes in their environment. Also, during the early life stages, maternal care is critical in shaping the stress response system. Kittens who receive a lot of maternal grooming and care develop more resilient stress responses, but those who do not get enough maternal care often have increased stress reactivity [73]. As cats develop, their stress reactions can be further influenced by their surroundings, including social interactions, territorial stability, and habits [18, 62]. How a cat acts when faced with a stressful stimulus depends on many individual factors [74, 75] and the perceived threat characteristics [76, 77].

As hunter–prey, cats are animals particularly predisposed to suffer the effects of stress, even related to small changes in their routines like feeding time variations or new furniture. These changes may trigger behaviors and symptoms of stress-related diseases (Table 2). Understanding the development of stress in cats is critical for increasing their welfare and effectively regulating stress-related behaviors.

## 4. Effects of Stress, Anxiety, and Fear on the Health and Welfare of Cats

Some animals suffer acute stress as a result of stimuli that should not be interpreted as harmful, such as loud noises, quick movements, unfamiliar environments, and strangers [62, 78]. This stress can limit exploration and play, reduce sleep, and increase hiding [82]. For example, cats exposed to stressful settings, even briefly, exhibit more immunosuppression [62] than those in stress-free environments [83]. Environmental variables like living in a multicat household can also contribute to immunosuppression, as these cats frequently have high red blood cell counts but low white blood cell counts. Also, stress in cats is linked [62] to organic conditions such as diarrhea, vomiting [80, 81, 84], feline idiopathic cystitis [62], hyperglycemia (acute stress) [85–87], and anorexia that leads to hepatic lipidosis [88]. Stress has a particularly negative impact on cats with feline interstitial cystitis (FIC) [89] worsening symptoms like increased urine frequency, painful urination, and recurrent cystitis, which significantly affect the cat's welfare [62]. Also, stress related to poorly enriched settings may lead to lower bladder permeability [62] and also elevated plasma norepinephrine and dihydroxyphenylglycol levels [90]. The above also explains how one of the most important tools to work on these problems in cats is environmental enrichment [78]. Like any interpretation of reality, not all individuals find the same stimuli stressful [91], so what is extremely distressing for one cat may not cause any discomfort for another.

Mariti et al. [92] found that anxiety in cats at veterinary clinics might cause a stress reaction that interferes with diagnosis and treatment, compromising their welfare. The clinical environment, unknown individuals or animals, and physical handling during examination are all considered significant stressors for the cat. Studies based on surveys have shown that stress is one of the reasons caretakers avoid taking cats to the veterinarian, or at least avoid doing so until the pathology is manifest, since they consider the trip, stay, and return home as highly negative experiences for the cat [92–94]. It is also important to remember that stress is cumulative in nature, so the more stressful experiences and factors a cat has, the more stress the situation causes [95]. This leads to a high percentage of these animals not receiving the veterinary care they need, since visits to the vet are associated with unpleasant and unfamiliar smells, annoying sounds, particularly suffering and pain. Since the cat's previous experiences with the vet are often linked to the above, many exhibit symptoms of acute stress in the exam room.

The stress response in cats can be seen in many different ways, as variable as the individual, but there are some that are much more common. Generally, the cat will try to evade a potential threat choosing to hide or display “freeze” behavior [96–98]. However, if the stimulus considered dangerous is very close, very large, or threatening, and fleeing is not allowed, it will opt for agonistic protective behavior (aggression) [76, 77]. It is important to consider that aggression is not the cat's first response to fear; on the contrary, when scared and faced with manipulation and contact, it will possibly show calm and docile and almost completely inhibited. Sometimes, the cat who appears to be “comfortable” and steady is in fact experiencing an elevated level of stress which causes him to exhibit “learned helplessness” [58]. When this happens, the animal appears to be calm, avoiding eye contact, tolerating procedures, and tolerating handling procedures that other cats would not; it is critical that when detecting these, the professional stops and assesses the situation considering the cat's emotional state [63, 71]. “Learned helplessness” should not be confused with “freeze” behavior, since, although their purpose is the same (to avoid physical violence), they arise from different emotional systems [99]. We can also find a patient with agonistic behavior, vocalizing, and displaying a clear protective reaction product of fear, pain, or fear of pain [100, 101]. Unfortunately, it is often challenging for caretakers to recognize subtle signs that cats may display as a result of stress [37]. Symptoms associated with stress manifest differently depending on the triggering situation and the individual [102, 103]. Cats exhibiting chronic stress may display pathological behaviors such as stereotypies (repetitive behaviors without a specific purpose), psychogenic alopecia (self-inflicted hair loss due to excessive licking, which may lead to injury), and even self-mutilation of the limbs and tail [104, 105]. Even though the signs seem to be quite clear, it must be considered that it is possible for a specific stimulus to induce stress in one cat but not another [102]. A number of factors influence these variations, the most important of which is the cat's “coping strategy” [106]. Stella and Croney [106] describe “coping strategy” as the strategies and actions that cats use to effectively deal with stress and anxiety-provoking events. Such strategies serve as adaptive mechanisms, allowing cats to cope with and lessen the detrimental impacts of stressors in their surroundings. Cats' coping style is influenced by a variety of elements, including the stressor's nature, cat temperament, previous experiences, and bond with their caretaker. Stella and Croney [106] discovered that cats, like other animals, employ a variety of coping strategies in response to stress, including both physical and behavioral reactions. These include concealment, avoidance of specific stimuli, antagonism, and enhanced attention.

### 4.1. Cat Caretakers and Feline Patients

In recent years, various studies have highlighted that the experience linked to a veterinary visit is highly stressful for both the cat and its caretaker [94, 107]. Studies based on surveys of caretakers found that the majority consider that their cat hates going to the vet (58.2%) and that the mere idea of taking their cat to the vet is stressful for themselves (37.6%). They also found that caretakers would take their cats more often if the experience was less stressful (28%) [93]. Other surveys showed that stress is the primary reason caretakers avoid or delay taking their cats to the vet until the condition becomes severe, since they consider the whole experience as highly negative for the cat [92, 93]. Researchers in the field had already established that cats show multiple signs of stress in the examination room [37, 42], and urge the veterinary community to train in the specific handling of the species. One reason identified is insufficient training concerning animal welfare and emotional health, which reflects in the quality of care provided to cats [108–110]. It was also found that cat caretakers frequently have deep emotional attachments to their cats, which is why they seek higher levels of care, follow more veterinarian recommendations, and are more prone to comply with prescribed treatments despite associated expenses [111]. Even though there have been interesting breakthroughs in feline medicine research in recent years, cats' emotional welfare has just lately emerged as a focus in overall welfare [15, 42, 110].

Regrettably, the lack of knowledge among cat caretakers and veterinary staff makes it even more complicated for cats [112]. In veterinary visits, cats' behavioral responses are often rooted in anxiety, fear, and stress, which can lead to unpleasant and occasionally dangerous circumstances, leaving caretakers feeling uncomfortable and even shamed by their cats' behavior. The emotional health model [38] is one approach developed to address this issue from a scientific and compassionate view. According to this theory, it is best to avoid characterizing these replies as “negative” while interacting with the caretaker (or general public). Instead, it is encouraged to refer to them as “protective behavioral responses” [42], since this language facilitates comprehension and empathy from both the caretaker and the veterinary team involved in the cat's care [38]. Also, this term highlights the evolutionary function which usually is protecting the individual from danger. This view helps decrease negative connotations that are sometimes associated with these agonistic behaviors during consultation, particularly in cats. Although it is essential to stay up-to-date and informed about therapies, discoveries, and management of feline patients, most caretakers do not evaluate veterinarians based on it, but rather on the interaction and handling, they have with their cat [65]. This emphasizes the importance of practicing respectful handling and interaction as an essential component of veterinary care.

We must consider that communication with the caretaker is essential in this field. Cats can discern the emotional condition of their conspecifics and humans, particularly their caretakers [113], and it makes it even more important to establish a respectful and positive bond with them. Assessing their bond, which is a complex interspecies dynamic, seems more complicated and important in the cat's behavior than previously thought [114]. For example, caretakers with a neurotic personality are likely to live with cats who exhibit greater levels of aggression, anxiety, and fear, often accompanied by other behavioral disorders [112]. These cats may suffer not just medical diseases as a result of stress but also psychological effects, especially if suitable coping mechanisms are absent or limited. The latter has motivated a deliberate attempt to urge veterinarians to undergo “Cat Friendly” interaction training and a considerable amount of research in cat interaction practices.

### 4.2. The “Cat Friendly” Professional

The evolution of new concepts and the shift toward respectful treatment of cats in clinical settings have spurred a surge in publications on this topic. Consequently, relevant institutions have developed certification programs tailored for professionals and clinics, encompassing interaction, handling, personnel, and infrastructure. The two leading global institutions in Feline Medicine, the ISFM and the AAFP, have released two “Cat Friendly” interaction and handling guidelines, which are freely accessible online [98, 115]. Currently, these two institutions organize and handle international programs for certifying clinics as “Cat Friendly,” which are actually two, depending on the geographic location: the Cat Friendly Practice® Program (supported by the AAFP in America) and the Cat Friendly Clinic Accreditation Program® (supported by the ISFM, in Europe, Asia, and Africa). These programs are specifically developed to assist veterinary clinics in better understanding the unique needs of cat patients and creating a friendly and respectful environment for both cats and their caretakers at the veterinary clinic [116]. A widespread campaign encourages veterinarians to undergo this “Cat Friendly” handling training, with the AAFP now also offering certifications in Spanish and Portuguese.

Another renowned institution that has gained popularity in recent years and is now regarded as a global authority on the subject is Fear Free. This institution is committed to improving domestic animal care by preventing and reducing fear, anxiety, and stress via caretaker education and inspiration. They adopted the concept of “FAS” (fear–anxiety–stress) as an ordered process in which these emotions often manifest, and they offer resources like online courses to help people identify and handle these emotions proactively. Fear Free focuses on reducing stress and discomfort for patients during veterinary visits, resulting in a positive experience for pets, caretakers, and the veterinary staff [117, 118].

## 5. Recommendations for a Successful Consultation With the Feline Patient

### 5.1. Considerations for the Vet Visit

One thing most authors agree on is that a successful veterinary visit starts at home. Preparing and training the cat previously is critical for success [119], beginning with educating the caretakers [92]. It is recommended that cats are always transported in cat carriers. It is critical to underline the importance of acclimating and training the cat to freely enter and exit the cat carrier [120]. Caretakers are recommended to keep the carrier open and available at home, near the cat's favorite resting locations, and to place little amounts of food inside to encourage acceptance [98]. Introducing the carrier exclusively during veterinarian appointments may result in negative associations; thus, it should also be associated with happy experiences, such as relaxation and feeding. Pheromones can be used inside the carrier (applied 15–30 min prior to allowing access to the cat) to promote a calming environment [72, 98, 100, 115, 121, 122]. Unfortunately, improper carrier handling and negative clinic experiences may lead cats to hide in the presence of the carrier [93] and vocalize during the journey to the clinic [93, 107]. One of the fundamental tools to work on this problem is classical conditioning and classical counterconditioning [71], which will allow the cat to positively associate the presence of the carrier with rewards, tranquility, and positive experiences. It is important to note that the cascade of stressful events that trigger a behavioral response of stress or fear in the clinic usually began long before arriving there. For example, in one study, more than half of the caretakers (51.5%) recognized signs of stress in their cat before leaving the home [92], and considering that caretakers do not usually detect minimal signs of stress in their cats, we can assume that the emotional state is probably worse than reported. The means of transportation also affect the cat's level of anxiety and stress since, like many mammals, including humans, they can present motion sickness that would only generate greater annoyance and discomfort [123]. Caretakers have described that their cats vomit, vocalize, and defecate on the way to the vet (behaviors that should not be underestimated) and that their perception is that the stress associated with the veterinary visit is a barrier to the success of the visit [124]. It is recommended to ask if the cat presents any of the symptoms, such as drooling, lip licking, or vomiting, in order to know if this patient would actually benefit from antiemetic treatment prior to the trip [98, 125]. The recommended medication to avoid this discomfort is maropitant (Cerenia™), widely used in daily clinical practice. It will be recommended at a dose of 1 mg/kg every 24 h, as it has been proven to be useful in avoiding motion sickness in cats prior to a veterinary appointment [98, 123, 126]. It should be administered 4 h before the trip in conjunction with 2–3 h of fasting for better treatment effectiveness, and there is no contraindication to the concomitant use of anxiolytics [98].

Even if the training is successful and the cat voluntarily enters the carrier, leaving home and the trip to the veterinarian can be negative experiences for the cat [94]. Signs of fear, anxiety, and stress may occur, such as reduced activity, freezing, ear flattening, climbing, heightened vigilance, spraying, altered elimination habits, decreased grooming, and changes in sleep patterns may occur [127]. It is known that the environment of the veterinary clinic itself causes changes in the physiological parameters in the cat [128], and they also suffer from “white coat syndrome” [129]. A study in 95 cats found that vocalizations and tachycardia (associated with mydriasis) were linked to stress and fear in the context of a visit to the veterinary. Another study, linked to behavioral responses in this environment, found a third of the cats had escape attempts [107], and elderly cats exhibited more fear or irritability than young cats. This difference is explained by the lack of negative or traumatic experiences related to the veterinarian from the young cat group [107] which highlights the importance of linking the vet visits with positive experiences and not just avoiding negative ones [38].

### 5.2. Best Practices for Feline Patient Arrival and Waiting Areas

Caretakers should book the vet visit ahead of time to ensure a successful experience [98, 115]. The clinic receptionist plays an important role at this stage because they are the first point of contact with the caretaker, acquiring potentially vital information for the success of the appointment. During the scheduling procedure, the receptionist will provide the caretaker with a questionnaire in which they will be asked about the cat's basic history details, like previous visits to the clinic, a history of transportation issues, and interactions with veterinary staff. For first-time patients, caretakers should inquire about the cat's previous vet visits and tolerance for handling, particularly by unknown people [98, 115]. This information will allow the veterinary team to adapt the appointment to the cat's individual needs [98, 115]. This is also an excellent opportunity to provide recommendations on cat carrier habituation, at-home handling techniques, and tools that can aid in a stress-free journey to the clinic appointment, since this will also impact the cat's experience at the clinic.

On the appointment day, the veterinary team should be alert upon the patient's arrival, assisting the caretaker by opening the clinic door and directing them to the waiting area, thus allowing the caretaker to transport the cat carrier on their forearms and not by the handle. Placing the carrier on raised surfaces and away from other cats reduces stress responses because elevated surfaces create a sense of security. It is advised that non–feline-exclusive clinics offer a separate physical location for cats, ideally equipped with at least one pheromone diffuser per room or per 70 m^2^ [72, 100, 115, 121, 122]. If it is not possible to have a dedicated waiting space for feline patients, the cat can be admitted directly to a free exam room while waiting, or different attending hours can be assigned for cats to avoid them sharing spaces with other species [17, 98]. Caretakers should bring a blanket or towel with a familiar scent to cover the carrier, minimizing external stimuli and light exposure, which is preferable for cats [17, 98, 115, 121]. If a caretaker lacks a blanket or towel, the veterinary staff should offer a clean one for each patient [17].

### 5.3. Physical Spaces and Environmental Considerations

The exam room where the cat will be cared for is of significant importance. When considering the exam table, it is important to avoid slippery, hard, and cold surfaces that can cause discomfort to the cat, as it may become frightened by reflections or shadows projected on it. To prevent these discomforts, nonslip mats, foam mats (such as a “yoga mat”), or towels and blankets (if provided by the clinic, a new, clean one should be used for each patient) can be used [130, 131]. Caretakers may be urged to bring their own blankets or towels, as the familiar scent may make the patient feel more at ease. Care in another setting may be feasible for some patients, such as home visits. Studies have shown that managing cats in their home environment can be challenging [128], particularly because the natural behavior of a carnivore in its territory tends to be defensive and aggressive. However, other studies recommend home visits [132]. A study comparing physiological parameters of cats cared for in a clinical setting versus at home (using low-stress techniques) found an increase in glucose levels and more attempts to seek hiding places in the clinical setting, with no significant differences in cortisol levels [132] Also, in cats cared for in both environments, it was discovered that those cared for in the clinic and subsequently at home had a significant decrease in cortisol levels, underlining the value of familiarity and maybe the application of low-stress strategies [132]. While the authors emphasize that the idea that a cat will be easier to manage at home due to lower stress than in a veterinary clinic is not generally applicable because the cat in its home is often exposed to other stimuli that cannot always be controlled (such as the presence of other animals). It is ultimately up to the veterinarian to decide whether such services will be provided.

As a crepuscular species, cats like low-light environments, and thus, dim illumination makes them feel safer and more calm. The use of perfumes, air fresheners, or strong scents in the examination room is discouraged [1, 133]. This is mainly because a substantial amount of the cat's brain is dedicated to olfactory perception, making them more sensitive to certain smells [1]. Olfactory stimuli are essential for cat communication and connection building, as well as their mental and physical wellness [60]. Facial pheromone analogs should be used whenever possible since they demonstrate to improve food intake, grooming, and facial rubbing, all of which are connected with a sense of safety and tranquility in the environment [60, 115, 134].

### 5.4. Patient Interaction and Handling Techniques

Once in the examination room, it is recommended that the cat be allowed to exit the carrier voluntarily by opening the carrier door and avoiding intruding into its space, making sudden movements, or direct eye contact. During the cat's acclimatization to the new environment, the veterinary staff will gather needed information to complete the clinical record, avoiding interaction with the cat unless solicited by it [17]. A thorough history should be taken, including questions regarding behavior, interactions with cohabitants and neighborhood cats, and environmental enrichment. During the anamnesis, try to inquire about environmental enrichment at home, including resources for self-preservation behaviors such as hiding spots to assess stressful situations without feeling observed [135, 134]. Keep in mind cats can visually distinguish between familiar and unfamiliar humans [136], recognize their caretaker's voice, respond to their own name, and use their caretaker's reactions in threatening situations to guide their own [113, 137], so the presence of an attachment figure benefits the cat and the veterinary team [98]. Furthermore, in a study that assessed the responses of cats to various favorite rewards (human social interaction, food, toy, and scent), it was discovered that 50% choose human social interaction over other incentives such as favorite food or a cherished toy [60]. This was the case for both cats with caretakers and shelter cats, and therefore, it is best to take advantage of the link with their caretaker to help the patient relax during the appointment. Also, we know that personal preference for what individual cats like as a stimulus to work for is crucial for properly influencing behavior [60, 138]. As a result, stimulating tools will be accessible in the exam room, such as food, toys, pheromones, and the presence of the caretaker as another stimulus that can help benefit the patient's emotional state. Consider the caretaker's emotional connection to the cat, especially when they report problematic behaviors, since problematic behaviors that directly impact their caretaker's lifestyle are more reported (e.g., excessive grooming, fear, phobias) [139].

Once finished with the anamnesis, if the cat does not voluntarily exit the carrier, it is advisable to conduct the examination with the patient inside of it if feasible, avoiding forceful removal. Cats often remain calmer when allowed to stay in the carrier during the examination; thus, carriers with a removable top half are preferred [115, 140]. In cases where the cat must be fully removed from the carrier, it is important not to tilt it, causing the cat to fall. Instead, if possible, attract the cat by calling it with food or toys, and if ineffective, gently support the cat's caudal abdomen and hind legs to encourage forward movement [115]. If the cat shows signs of fear and anxiety and must be taken from the carrier, remove the top of the carrier while covering the exposed edge with a towel or thick blanket to provide the cat with a sense of security [115]. Throughout the process, it is critical to analyze the cat's body language and vocalizations, as these reveal significant information about the cat's emotional state. When handled or constrained, cats may exhibit anxiety behaviors such as lip licking, swallowing, excessive salivation, reducing their apparent surface area, placing their limbs under the body, wrapping their tail against it, positioning the ears to the sides or back of the head, dilation of the pupils, and tail movements [96, 98, 127].

### 5.5. Low-Stress Handling and Restraint

A few years ago, physical restraint was regularly employed in clinical settings with the primary goal of ensuring the healthcare professional's safety and optimizing appointment times. However, it is now understood that physical restriction is not an appropriate method of handling these patients. Modern techniques that prioritize the welfare of the cat and result in reduced physical risk for the professional are now encouraged [141]. By employing respectful “cat-friendly” interaction based on modern information of feline behavior, we can enhance the health of our patients, strengthen the bond with their caretakers, and promote the welfare of both humans and cats [17, 141]. The objectives of “cat-friendly” and Fear Free handling techniques include minimizing fear and pain, enhancing the veterinarian–client–cat relationship and trust (leading to improved lifelong medical care for the cat), increasing efficiency, productivity, and job satisfaction among the veterinary team, as well as facilitating prompt identification and early intervention for medical and behavioral issues, among other benefits [115, 118, 142]. It is now widely accepted that all veterinarians should consider implementing low-stress handling techniques that are respectful and considered to the feline species [37].

For physical handling, it is advised not to use tools such as cat bags, nets, and gloves; if a cat displays prolonged resistance (more than 3 s) and/or signs of fear or stress, alternative methods like chemical restraint should be considered to aid in management and ensure respectful treatment of the patient, preventing escalation of FAS responses [115]. Although direct physical hostility is usually a last resort, the cat may display defensive aggression when unsuccessful with avoiding perceived danger. Defensive aggression in cats is characterized by a variety of physical indicators, including high-intensity vocalizations like growling, shouting, defensive meowing (yowling), hissing, and spitting. Cats may adopt a crouching position to appear larger, frequently accompanied by a piloerection [17, 98], and may attempt to attack by scratching and/or biting [17]. Professionals need to focus on counterconditioning and positive conditioning of feline patients to prevent instances where aggressive behavior is displayed, and to avoid situations where cats resort to direct aggression when contact with veterinary staff is attempted after previous successful avoidance [100].

Once out of the carrier, it is recommended that cats be allowed to walk freely and approach voluntarily to give them a sense of control. During the physical examination, cats should be allowed to select their preferred position and location in the room for comfort, which may not be on the examination table, exhibiting flexibility in this regard. This strategy alleviates tension by empowering the cat with a sense of control. An appropriate species-specific approach considers a slow-paced approach avoiding direct eye contact, positioning yourself at the same level as the cat, and refraining from leaning over [17, 115, 128, 131, 143]. Keeping a moderate to low speaking volume, avoiding excessive loudness and sounds that cats may perceive as threatening, such as “shh,” is recommended, as these sounds can elicit fear responses [115, 144]. Other science-based strategies include playing cat-specific music that has been shown to help in reducing stress and improve care quality [145].

At first, efforts should be made to evaluate the cat without physical contact. If the cat tolerates or seeks contact, it is advisable to allow it while continuing the evaluation without restricting movement unless absolutely necessary, and even then, for a maximum of 2–3 s [96, 119, 146]. Once safe physical contact has been established and the cat is comfortable with handling, the physical examination can begin. Passive wrapping techniques with towels or blankets are preferred over excessive restraint, as this method, in contrast to traditional practices, results in a significantly calmer, more practical, and effective approach in feline clinical management [141]. Observing the patient's body language and communication cues is critical to determine when to provide space, pausing the examination briefly to improve tolerance to contact and manipulation [115, 128, 143]. Scruffing, a common maneuver in traditional practice involving holding a cat by the dorsal neck area to control movement, is no longer recommended and is discouraged by reputable institutions advocating for empathetic and respectful patient handling (AAFM and ISFM), as well as organizations promoting respectful practices (Fear Free™). Scruffing is known to enhance arousal and fear in cats, compromising their sense of control and welfare, all of which are important considerations in feline care [147]. Recent research has demonstrated that both clips esthesia (placing clips in the nuchal fold) and scruffing are similarly distressing for cats, resulting in higher stress-related behaviors and thus aversion [98, 115, 147]. Complete physical restraint is also discouraged [98, 115, 146]. Employing a slow and confident approach with gentle and deliberate movements is the most effective and suitable method for handling patients during examination [128, 131, 143].

### 5.6. Environmental Control and Stress Reduction

Ideally, cats should not be moved between rooms for different procedures or tests, requiring careful planning to have all materials and equipment in the same examination room to prevent fear and stress associated with entering and exiting the examination room. It is recommended to maintain indirect eye contact, move slowly and purposefully, and minimize hand motions during clinical examination. Effectively controlling interactions with cats is critical for their physical and mental welfare, although performing a comprehensive medical examination can be difficult in anxious patients. According to one study, 66.7% of veterinarians experienced difficulty indirectly monitoring blood pressure in feline patients because of cats' high levels of fear, anxiety, and stress [148]. Consider that the head and neck are preferred areas for physical contact, so gentle touching, particularly the cheeks, as well as soft palpation and manipulation techniques, can promote tranquility and comfort while also allowing contact with the cat's facial pheromones [115, 125]. Remember that signs like purring, which was previously related to calm and pleasure, are now acknowledged to be associated with a variety of stimuli, both positive and negative (such as pain) [17]; therefore, purring as an isolated sign should not be seen as a reaction to a successful handling.

It is advisable to have a variety of treats available for the cat, including palatable food (tools like LickiMat can be used for hands-free interaction), toys (especially for kittens), and cat grass, to engage cats positively throughout the examination [100, 115]. Implementing palatable rewards and emotional reinforcement to encourage desired behaviors not only strengthens the bond with the veterinarian but also makes the patient's experience a lot better [115, 128, 131, 141]. Although an excellent enrichment tool in other situations, catnip is not recommended during the veterinary examination (except after finishing) as it can cause hyperarousal and potentially lead to aggression in some patients.

### 5.7. Feline Ethology Consultation at the Veterinary Clinic

A feline veterinary ethology professional is a specialist that through veterinary science and ethological principle will try to discover the underlying reasons for behavioral issues. The first and most important thing is to try to establish whether the behavioral cause of the consultation is the product of something organic or is of behavioral origin. Many organic diseases present behavioral signs [149–154] (discussed later). The consultation process is often divided into four distinct phases which includes an initial assessment, a diagnosis, an intervention, and a follow-up.

The first phase is the initial assessment. The initial examination is critical for getting detailed information about the cat's behavior. During this phase, the veterinarian or veterinary behaviorist gathers data using a variety of methods. Caretakers are frequently asked to complete a lengthy questionnaire on their cat's history, the environment, daily routines, previous experiences, and specific behaviors of concern [71]. This is supplemented with direct observation of the cat, preferably in its own environment, to avoid stress responses and to better understand its natural habits. Video recordings provided by the caretaker may also be useful for this.

In the second phase, professionals will try to get a diagnosis. Following the data collection and assessment, the professional provides a diagnosis. This method involves determining whether the behavior is normal for the species, a reaction to external stressors, or a sign of an underlying medical issue. For example, urinating in an adequate place (formerly known as “improper urination”) could be caused by a urinary tract infection rather than behavioral difficulties [155]. This phase may require more medical testing to exclude or confirm health concerns, which is critical for developing an effective intervention plan.

The third phase is an intervention phase. This phase adapts to the cat's unique requirements and most likely consists of medical treatment, behavioral modification methods, and environmental alterations. The most widely used approaches are positive reinforcement, environmental enrichment (such as hiding places and interactive feeding puzzles), and pheromone therapy [156]. If medical problems are identified, appropriate treatments are prescribed. The objective is to address both the symptoms and the underlying causes of the behavioral issues.

The fourth phase consists of a follow-up. The concluding phase involves monitoring the cat's progress and making modifications to the intervention plan as necessary. Follow-up consultations are arranged to evaluate the effectiveness of the interventions and to ensure the cat and its caretaker are adapting well to the changes. These sessions facilitate ongoing support and adjustments, which are frequently required as the cat's behavior and environmental influences evolve. So, feline veterinary ethology entails an integrated approach to diagnosing and treating cat behavior problems, considering physiological, psychological, and environmental factors. The effectiveness of this method is dependent on a thorough initial examination, accurate diagnosis, personalized therapies, and rigorous follow-up. This must be completed by a skilled and up-to-date professional.

### 5.8. Main Organic Causes of Behavioral Problems in Cats

Cats, like any other animal, can experience behavioral abnormalities that are generally caused by underlying organic issues (Table 3). The first step in identifying a behavioral condition is to rule out any physical factors. These can cause a cat's behavior to change, potentially triggering problems or concerns for its caretakers. Understanding the relationship between physical health and behavior is critical as many of these can be subtle and develop gradually. It is important to recognize that such habits are not just erratic behaviors, but may also be symptoms of health issues that require medical attention. In order to address them with an effective approach, it can be helpful to have a general awareness of how different organic conditions influence behavior before looking into specific conditions. Each of these possible reasons for behavioral changes demands a thorough veterinary evaluation. Identifying them as potential causes of behavioral abnormalities allows for more accurate and efficient treatment, increasing the likelihood of improved welfare for affected cats and their caretakers.

### 5.9. The Importance of a Multimodal Approach

After organic problems have been ruled out, the focus changes to treating any identified abnormal behavior. The most successful method for stress management in felines is multimodal therapy, which involves environmental enrichment, pheromones, psychotropic pharmacological support, and caretaker orientation [98, 125]. To establish a framework for the necessary environmental enrichment for the physical and mental welfare of cats, the American Association of Feline Practitioners (AAFP) and the International Society of Feline Medicine (ISFM), developed the “5 pillars” of feline welfare [158]. These five principles intend to address the specific needs of cats at home and include the following:

- Providing an adequate setting for cats to feel comfortable and secure, such as holed cardboard boxes or hiding spots on high surfaces.
- Providing access to key resources in the appropriate distribution, such as water, food, litter boxes, scratching posts, play areas, rest places, and areas to sleep.
- Promoting play and normal predatory behavior.
- Motivating predictable, consistent, positive social human–cat interactions.
- Generating an environment that respects the cat's sense of smell.

Changes in or absence of any of the aforementioned principles may result in unwanted behaviors, affecting the relationship with their caretakers and potentially triggering fear, anxiety, and stress [82, 105, 159].

### 5.10. Pharmacological Tools for “Cat Friendly” Handling: Not the Last Option

Today, veterinary professionals have at their disposal a diverse range of drugs and nutraceuticals that can be employed for support, prevention, and treatment of the consequences of fear, anxiety, and stress while addressing the root cause of the issue. Gabapentin, fluoxetine, and lorazepam are examples of regularly used medications that are still used empirically in felines (they are not FDA-approved yet) [160]. Although some useful drugs, such as pregabalin, have recently been licensed for use in cats [161], others, such as fluoxetine, which is licensed to treat canine anxiety, have not yet been approved for use in cats [162]. For empirical drugs use, dosages are generally based on anecdotal evidence or the veterinarian's personal experience [163]. It is essential to bear in mind that while products and drugs are available, treatments for various behavioral disorders generally encompass environmental enrichment, training, and behavioral therapy, apart from pharmacological intervention. The best and most efficient technique combines these aspects, which has been shown to produce better results than the use of single drugs. Previous research has demonstrated that multimodal therapy is not only more effective [164], but it also produces faster outcomes [165]. Before beginning any drug treatment, particularly for behavioral modification, it is recommended to follow a set of criteria [166]:

- Perform a comprehensive physical examination and make a list of potential diseases if more than one is expected.
- Understanding about the neurochemistry of the presumed diagnosis(es).
- Understand the basic mechanism of action of the chosen active compound.
- Understanding the potential side effects.
- Ensure that the caretaker knows the expected changes once the effects of treatment start showing. The caretaker's involvement is key for treatment to be successful.

Medications also provide support to be able to perform a respectful physical examination if the cat cannot be re-coordinated and handling is necessary but without stressing out a patient who is probably already experiencing fear and anxiety. Chemical restraint is best used preventatively, as its effectiveness decreases as a cat's fear, anxiety, and stress levels rise. Veterinary personnel should be taught to recognize indicators of increased fear, anxiety, and stress in felines at the clinic in order to decide the proper timing and specific patients that require pharmacological restraint. Pharmacological handling or chemical restraint serves as an important tool that can enhance safety and reduce stress for the cat, the caretaker, and the veterinary team [100, 115]. It is crucial to recognize that fear is a primary trigger of aggression in cats at the veterinary visit [115], which is why every measure to reduce fear in cats in such situations should be taken. The ISFM and AAFP management guidelines [115] recommend the use of chemical restraint in the following circumstances:

- When a cat displays escalating fear, anxiety, and stress, or direct signs of aggression.
- When the patient's emotional condition may cause the treatment to take longer.
- When pain and suffering are expected.
- When mild restraint and appropriate equipment do not provide adequate safety for the veterinary team.

To mitigate negative emotions linked to anxiety during veterinary visits, it is recommended to administer anxiolytic drugs, such as gabapentin or pregabalin, at home to help reduce stress and improve the overall experience for the cat [124, 167–169]. If the cat shows signs of motion sickness or nausea during travel, antiemetic drugs like maropitant can also be beneficial [98, 123, 126]. Additionally, combining these anxiolytics with other medications, such as Trazodone, may further enhance the cat's tolerance to handling and reduce anxiety [170–172]. Other pharmacological tools include low doses of dexmedetomidine, opioids, or benzodiazepines. The choice of drugs and their combinations should be based on the specific needs of the patient and the procedure requirements. Chemical restraint protocols may vary depending on the attending veterinarian and their expertise, though consulting with a veterinary anesthetist is recommended for sound guidance given the continuous evolution and validation of new protocols [100]. The consideration of general anesthesia should arise if sedation proves insufficient for necessary procedures; however, subjecting the patient to unnecessary anesthesia should always be avoided if feasible, opting instead to schedule a new appointment under improved conditions (medication at home and better handling) to retry the procedure [115].

When gabapentin or pregabalin is not enough and other drugs such as trazodone cannot be used or are not chosen, a friendly sedation protocol can be implemented. One of the most commonly used protocols is based on that of Moffat [100]:

1. Low-dose dexmedetomidine. Dexmedetomidine binds to alpha-2 adrenergic receptors in the CNS, inhibiting the release of norepinephrine, leading to sedation and analgesia. The reversal agent for dexmedetomidine is atipamezole [173]. Consider that this reversal agent acts by counteracting the sedative and also analgesic effects of dexmedetomidine [174].
2. An opioid: frequently coupled with low-dose dexmedetomidine. The selection of the opioid should be based on the required level of sedation and analgesia [100]. It should be noted that mu receptor agonist opioids, such as morphine, are reversible (with naloxone), while other medicines, such as butorphanol, are more short-acting and ideal for quick procedures [115].
3. A benzodiazepine: for example, lorazepam or midazolam. These are recommended due to its hypnotic, sedative, muscle relaxant, and potential amnestic effects (also reversible) [100, 175]. Due to its reported hepatotoxic effects, it is advised to avoid the use of diazepam in this species [176]. The reversal agent for this group is flumazenil [177]. Administration of this reversal agent requires vigilant monitoring of the patient postadministration due to potential side effects [175, 177].

If it is determined that adequate sedation was not achieved with the previous protocol, minimal doses of ketamine can be added [100], which also provides a good analgesic effect [178–180]. Ketamine does not have a specific reversal agent, but atipamezole has shown effectiveness in reversing the combination of midazolam, medetomidine, and ketamine [175].

### 5.11. Behavioral Pharmacological and Nutraceutical Treatments

When addressing behavioral disorders in cats, a multimodal approach is frequently the most efficient. Among the numerous tools available, pharmaceutical and nutraceutical treatments show great potential. Pharmacological therapies include drugs that can affect brain chemistry and may help regulate behaviors including anxiety or aggression. Nutraceutical therapies, on the other hand, make use of natural ingredients such as amino acids, minerals, and natural supplements to improve behavioral issues. Both techniques seek to help cats and their caretakers by creating a more calm and stress-free environment. To classify the drugs most typically used to modify behavior related to fear, anxiety, and stress in cats, we will turn to Dodman [180], who classified them as follows.

#### 5.11.1. Dopaminergic

Selegiline is a medicine that, in low dosages, specifically and irreversibly inhibits monoamine oxidase B (MAO-B) while having minimal anticholinergic effects. However, at greater doses, it inhibits monoamine oxidase A (MAO-A), reducing its selectivity. Selegiline is widely prescribed for its impact on brain chemistry, particularly in Parkinson's disease, Alzheimer's disease, and some behavioral problems [181, 182]. Selegiline enhances the transmission of catecholamines and is approved for use in North America for cognitive dysfunction and in Europe for emotional disorders [163]. Encouraging results have been observed with selegiline in the treatment of feline fears, with a very low incidence of reported side effects, although pretreatment evaluation remains crucial [183]. When a cat displays fearful behavior toward recognized stimuli, therapy with selegiline at a dose of 1.0 mg/kg once daily is indicated [163]. For situations of aggressive reactivity to less specific stressful stimuli, clomipramine at 0.25–0.5 mg/kg once a day or fluoxetine at 0.5 mg/kg once daily may be appropriate [163].

#### 5.11.2. Noradrenergic/Serotonergic: Tricyclic Antidepressants (TCAs)

These drugs reduce serotonin and norepinephrine reuptake while also exhibiting anticholinergic, antihistamine, and alpha-adrenergic activity to varying degrees. TCAs are not recommended for individuals with heart disease, glaucoma, or urine retention [142]. Clomipramine, the most selective serotonin reuptake inhibitor (SSRI) among TCAs, also inhibits norepinephrine reuptake and has mild anticholinergic and antihistamine effects. Clomipramine is approved in Australia for separation anxiety in conjunction with a behavior modification program (BMP), anxiety disorders, and urine marking in cats [142, 184]. It has also been effective in treating feline compulsive behaviors. Prolonged administration may be required in some cases [142].

#### 5.11.3. SSRI

These drugs primarily inhibit the reuptake of 5HT1A in presynaptic neurons. Because of their unique impact on serotonin, they may have fewer negative effects than TCAs. Possible side effects may include increased intraocular pressure, sedation, or anticholinergic effects, but they do not have cardiovascular effects or lead to urine retention like TCAs. Paroxetine exhibits mild anticholinergic effects. The most common side effects observed are lethargy and anorexia. Fluoxetine and sertraline are effective in treating compulsive disorders, while fluoxetine and paroxetine may be beneficial for generalized anxiety. Fluoxetine may also be effective in managing feline urine marking and compulsive disorders [142]. Therapeutic regimens involving these medications are typically prolonged as the effects usually manifest after 4–8 weeks. As a result, it is critical to communicate with the caretaker about the timelines to be followed during therapy. Due to the danger of developing serotonin syndrome, these drugs should not be used together with MAO inhibitors (selegiline) or amitraz and should be prescribed with caution to epileptic cats.

#### 5.11.4. Selective Serotonin and Norepinephrine Reuptake Inhibitors (SNRIs)

Venlafaxine is classified as a selective SNRI and is another type of antidepressant that has received attention and shown promising outcomes [185]. Its dual method of action makes it an appealing alternative, even though it has a larger affinity for serotonin transporters than norepinephrine transporters [185, 186]. Venlafaxine is recommended for cats diagnosed with aggression and anxiety [187], and conditions often triggered by inappropriate play behavior. It is also indicated for the treatment of feline idiopathic cystitis (1–2 mg/kg, orally) [188]. For redirected aggression and anxiety, authors suggest its use due to its efficacy and minimal side effects, especially in patients intolerant for selective serotonin reuptake inhibitors. The recommended oral dose is 1–1.1 mg/kg [187, 189], and its practical application is highlighted in the literature as the drug capsule contains granules that can be easily administered with a palatable vehicle (food) [189].

#### 5.11.5. Serotonin (5HT1A) Receptor Agonist and a Dopamine (D2) Agonist

Buspirone may be beneficial for mild fear and anxiety [190], for urine marking in cats [191], and, in one report, for psychogenic alopecia [192]. It does not induce sedation, stimulate appetite, or impair memory. The onset of its effects may require between 10 and 14 days to become evident. It is important to note that, like any anxiolytic, it has the potential to induce aggression due to disinhibition [142]. The recommended dose for cats is 0.5–1 mg/kg, once or twice daily, with a maximum of 7.5 mg/cat twice daily [190].

#### 5.11.6. Serotonin Antagonist and Reuptake Inhibitor (SARI) and Alpha-1 Adrenergic Receptor Antagonism

Trazodone is a SARI which is regularly prescribed in human medicine as an antidepressant, anxiolytic, and hypnotic agent [193]. It has been used safely in canines, so in the last decade, publications began regarding its use in cats with excellent results to help them tolerate travel, waiting, and handling during the consultation [170–172]. Its effect is dose-dependent [194] and does not generate changes in echocardiographic variables, and although it slightly decreases blood pressure, it does not decrease heart rate [195]. In one study, no synergistic effect was found between gabapentin and oral trazodone, although the number of cats in the study was quite small (*N* = 6) [196]. The recommended empirical dose of trazodone is 50 mg/cat orally, 120 min before leaving home, although in felines, it has a wide safety margin. Studies have been published using doses of 100 mg/cat orally without side effects in patients who exhibited lower levels of FAS (compared placebo) [170]. It has also been used transdermally at a dose of 150 mg/cat with lowering stress and anxiety during transportation in cats [197]. Something to consider is that most owners give positive feedback to the transdermal method of trazodone administration [197], so it would be good to make it more accessible in countries where it is still difficult to obtain, such as in Uruguay.

#### 5.11.7. GABAergic Agents

Benzodiazepines function by enhancing GABA, the primary inhibitory neurotransmitter of the nervous system. These psychotropic substances are used to treat fear and anxiety by promoting muscle relaxation and reducing locomotor activity. They are also used as appetite stimulants because of their ability to cause hyperphagia although they are rarely effective in critically ill cats [198]. Benzodiazepines can cause paradoxical excitability and may impair learning and memory [190]. The use of diazepam is not recommended as a first-line treatment for felines if alternative medications are available, considering reports of hepatotoxicity and subsequent fatalities [176]. Given that lorazepam lacks active intermediate metabolites, it is generally recommended for feline use and is a suitable option for patients with compromised liver function. The recommended dose of lorazepam for cats is 0.125–0.25 mg/cat, once or twice daily [190]. Benzodiazepines achieve peak effectiveness shortly after each administration and are therefore employed for their short-term effects [142].

#### 5.11.8. Anticonvulsants

Within this category, gabapentin and pregabalin are included not by formal classification but due to the effects they elicit in cats. Gabapentin was initially created as an anticonvulsant, serving as a structural analog of the inhibitory neurotransmitter γ-aminobutyric acid (GABA). It has GABA mimetic capabilities and can pass through the blood–brain barrier. Until today, the mechanism of action remains uncertain [199]. This medication has a variety of applications in veterinary medicine, including anticonvulsants, analgesics for chronic pain, and anxiolytics, with the last one being the one that interests us. It is widely used as a premedication for anxiety in both house and shelter cats [86, 98, 124, 167, 200]. Its popularity has increased dramatically in North America and Europe, thanks to more user-friendly formulations for feline administration. Furthermore, it has been shown to be as effective in increasing appetite as mirtazapine [200], making it a viable option for individuals suffering from anorexia who require both anxiolytic and appetite-stimulating effects. In research with shelter cats, both gabapentin and pregabalin were successfully used concurrently, with transdermal mirtazapine facilitating the cats' voluntary ingestion of gabapentin (offered with food) [201]. These findings are significant as they enable medication administration in cats that are difficult to medicate safely with minimal restraint (community and shelter cats), subsequently allowing for physical examinations or sedation for invasive procedures such as neutering without compromising the cats' welfare. Common side effects include ataxia, salivation, and sedation (despite not being a sedative) [98, 202]. Given that renal excretion is the exclusive route of metabolism in cats, dosage adjustments are necessary in patients with associated renal diseases [203] without avoiding its use [98]. Furthermore, although typically administered as single or occasionally double doses for premedication (the night before and 2 h before the veterinary visit) [98], gabapentin is now being investigated for chronic administration as an anxiolytic with promising outcomes [204]. The premedication dose of gabapentin for veterinary visits ranges from 50 to 200 mg/cat, with 100 mg/cat being the usual recommendation, administered 90–120 min before the stressful event (e.g., veterinary visit and car ride) [124, 167–169]. The most frequently observed side effects are salivation and ataxia [167]. The administration of gabapentin does not impact echocardiographic parameters or physiological variables [205] but may affect certain aspects of the neurological and musculoskeletal examination [206]. Pregabalin, another viable option, has garnered substantial evidence in recent years and, at varying doses, produces an effect very similar to gabapentin (with the advantage of lower dosage formulations in Latin America, making administration in cats more convenient). The recommended dose of pregabalin ranges from 2.5 to 7.5 mg/kg, administered 60–120 min before the stressful event (similar to gabapentin) [207, 208]. Also, both gabapentin and pregabalin are effective perioperative sedatives [209].

#### 5.11.9. Modulators of the Endocannabinoid System

Recent research suggests that CBD may significantly reduce stress in cats. In a study [210] (currently under review), CBD was administered as a sole treatment, for two groups of cats subjected to a validated thunder simulation. CBD was administered to the animals for 15 days at a dose of 4.0 mg/kg/day, indicating that it may have a benefit in reducing acute stress associated with veterinary visits. Nonetheless, research into this compound and its potential applications continues.

Keep in mind that chronic administration of behavior-modifying drugs should be indicated by a veterinary specialist, accompanied by a close follow-up and, if needed, behavior modification techniques (such as desensitization and counterconditioning) [37]. It is also critical to educate and work closely with the cat caretaker to improve the patient's adherence to the treatment. A clear definition of the expected length of effects for each therapy is required, as false expectations from the pet caretaker can arise. A study looking into pet caretakers willingness to administer drug treatments to their cats for behavioral disorders produced interesting results. Caretakers who had previously received psychiatric drug treatment were more likely to consider medicating their cats for behavioral issues [211]. Caretakers were more willing to administer treatment for specific behavioral concerns of their cats than for long-term treatment [211]. This emphasizes the need for skilled and open communication between the veterinarian and the cat caretaker. It is critical to set realistic goals and continuous assistance is critical for the long-term success of any pharmaceutical intervention, especially in addressing behavioral issues. Also, in addition to a comprehensive physical examination and thorough clinical review, it is advisable to conduct at least a blood chemistry profile (including renal and hepatic parameters), considering that the metabolism of psychotropic drugs typically involves the renal or hepatic systems [166].

### 5.12. Nutraceuticals and Supplements

It is important to consider that products labeled as “natural” that are not distributed by a licensed manufacturer may exhibit significant variations in purity, quality, level of activity, and, consequently, effectiveness and therefore should be avoided whenever possible. Factors such as adverse effects, toxicity, and contraindications should also be taken into account. Despite their current popularity, there is limited scientific evidence supporting some of the therapies falling into this category. Some examples include as follows.

### 5.13. Nutraceuticals Affecting Neurotransmitter Synthesis

#### 5.13.1. GABAergic Agents: Alpha-Casozepine

This compound is derived from the tryptic hydrolysis of bovine casein and has been suggested to possess anxiolytic properties similar to benzodiazepines, according to certain studies [142]. In a multicenter, randomized, double-blind, placebo-controlled trial, cats treated with alpha-casozepine exhibited increased contact with both familiar and unfamiliar individuals, along with an improvement (in this case, a decrease) in fearful and hiding-seeking behavior, as well as associated autonomic signs. As a result, the authors recommend its use in managing socially stressful conditions in cats [212].

#### 5.13.2. L-Tryptophan

This α-amino acid, naturally present in red meat, eggs, and dairy products, is utilized in protein biosynthesis. Due to its conversion to 5-hydroxytryptophan (5-HTP), a precursor to serotonin, it has been theorized that its consumption may alleviate symptoms of depression by increasing serotonin levels. The effects of supplementation have been studied in a variety of species, including poultry [213], pigs [214], dogs [215, 216], and cats [216], with varying results. The actual efficacy of L-tryptophan is questioned by most authors, as many studies have been conducted with small sample sizes and may not be universally applicable to all patient groups [217]. Limited studies have been published on its use in felines, despite its widespread clinical use in northern countries. In one study, cats administered L-tryptophan showed a significant reduction in stereotypies, vocalizations, agonistic behavior, and exploration. Additionally, elimination in undesirable places, furniture scratching, and agonistic interactions within the group decreased significantly [216]. Although the study used a small sample size (25 felines), the efficacy of L-tryptophan remains debatable. A commercial meal including L-tryptophan and alpha-casozepine reduced anxiety and fear-related indicators in a group of cats [117], but it is unclear if the effects were caused by one component, the other, or both combined. A similar study in dogs yielded comparable results [218].

### 5.14. Nutraceuticals Affecting Neurotransmitter Balance

#### 5.14.1. L-Theanine

This amino acid, highly bioavailable in tea, can traverse the blood–brain barrier in mammals in a dose-dependent manner within the first hour postadministration, persisting in the plasma and brain for several hours [219]. L-Theanine may be a useful oral dietary supplement in cats, considering its safety and convenience of administration to felines exhibiting apparent signs of stress (e.g., hypervigilance, mydriasis, excessive meowing, improper elimination). However, more research is required to prove its efficacy and optimal dosage [142].

#### 5.14.2. Chronobiotic Agents

Melatonin, derived from serotonin, can suppress dopamine and has sedative, hypnotic, analgesic, anti-inflammatory, antioxidant, and chronobiotic properties, making it an interesting premedication choice. Recent studies have investigated its anxiolytic effects at the preoperative level in people [220] and dogs [221], with encouraging results. While it has traditionally been used to suppress estrus in cats, continuing research is looking into its potential as an anxiolytic medication to improve compliance scores, with significant results without the ataxia seen with other treatments such as gabapentin [222]. The suggested melatonin dosage for cats is 0.65 mg/kg administered in a single dose [222].

#### 5.14.3. Pheromone-Based Therapies

Pheromones are semiochemicals used by animals for communication and have an important role in inducing specific responses in behavior [37, 222]. Pheromones are species-specific; each animal perceives those of others of the same species, but not those of others [223, 224]. Carnivores have been recognized for having the most developed and varied pheromone-secreting glands among mammals [223]. There are seven different kinds of pheromones in the cat, and the function of five of them are currently identified. Among these, facial pheromones, notably F3 and feline-appeasing pheromone, are extensively marketed worldwide. Feline facial pheromone fraction F3 (Feliway Classic®-Ceva, although other laboratories have produced other F3 analogs) is promoted as a tool for reducing stress and enhancing calmness [224, 225]. Cats often release F3 pheromone through facial rubbing to mark their territories and provide themselves with emotional stability [223]. The perception of this pheromone gives cats a sense of comfort and relaxation, lowering conflicts and aggression [224]. Synthetic F3 analog has been shown to help reduce furniture scratching, anxiety during travel, and veterinary visits and to facilitate introductions to new situations [163]. Additionally, an analog of another pheromone, feline-appeasing pheromone (Felifriends® or Feliway MultiCat®-Ceva), is specifically marketed for aggression-related situations and recommended for preventing intraspecific aggression when introducing a new feline to a resident cat [224, 225]. It has also been shown to reduce signs of anxiety and fear [226] and, in a study, reduced the number of episodes of cystitis in diagnosed cats [227].

## 6. Conclusions

Fear, anxiety, and stress are complex and significantly impact the health and welfare of cats. Their unique nature as pets and the expectations of their caretakers require veterinary professionals to understand the species-specific features of this animal population. When addressing physical and emotional welfare, patients must be approached through a comprehensive strategy that includes the cat's home environment, family interactions, and cohabiting animals. For veterinary visits, it is fundamental to work on training and handling strategies before traveling, during visits, and include feline-friendly interaction and handling to reduce fear, anxiety, and stress. Professionals must consider that pharmaceutical and nutraceutical interventions are effective in this matter, particularly during veterinary appointments. Careful selection of these active principles based on the individual cat is crucial for assuring welfare and treatment outcomes. This review presented and assessed a set of recommended strategies for managing fear, anxiety, and stress in domestic cats, as described in the literature. Continuous education for caretakers and the integration of evidence-based treatments into veterinary care are crucial for increasing feline welfare and improving quality of life.

## Figures and Tables

**Table 1:** Summary of cat's positive and negative emotional motivations. Adapted from Panksepp [41] & Heath [38].

Positive emotional motivations	Negative emotional motivations
“Desire-search” system: Search for essential resources	“Frustration” system: Triggered when the animal fails to meet your expectations
“Social game” system: Knowledge of one's own social competence and relationships with others	“Fear–anxiety” system: It distances you from danger and limits your response, ideally to flight, avoiding physical aggression
“Lust” system: Reproductive needs, mate selection, courtship, etc.	“Pain” system: maintenance of physical and functional integrity
“Care” system: mother–kitten bond	“Panic-grief” system: basically in kittens, it serves for the genetic survival of the species

**Table 2:** This table includes sickness behaviors in cats linked to stress. Grooming, social interaction, vocalization, elimination behaviors, and physiological indicators such as anorexia, vomiting, and lethargy are all examples of behavioral alterations. The studies also highlight behavioral reactions such as hiding, aggressiveness, and decreased vigilance in both healthy cats and those suffering from specific conditions such as feline idiopathic cystitis (FIC).

Research paper source	Sickness behavior described
Buffington et al. [78]	Urinary issues (e.g., frequent urination, blood in urine)
	Changes in grooming habits
	Vocalization changes
	Reduced interest in social interaction
	Weight loss

Kry and Casey [79]	Excessive grooming or self-mutilation
	Hiding or avoidance behavior
	Changes in litter box habits
	Increased vocalization
	Aggression toward humans or other animals

Rodan [17]	Inappropriate elimination
	Loss of appetite
	Increased sleeping
	Reduced play behavior
	Excessive scratching or marking

Stella et al. [80]	Healthy cats and cats with FIC:
	Anorexia/decreased food and/or water intake
	Vomiting
	Inappropriate elimination
	Fever
	Lethargy
	Drowsiness
	Decreased self-grooming
	Decreased social interactions

Overall [72]	Anorexia/decreased food intake
	Vomiting
	Diarrhea
	Lethargy
	Decreased grooming
	Increased fearfulness

Skånberg [81]	Dilated pupils
	Ears erected to front
	Partially flattened ears
	No vocalization/quiet
	Motionless alert activity
	Hiding
	Withdrawal behavior

**Table 3:** Overview of organic systems in cats and their associated symptoms and behavioral issues.

Affected organic system	Behavioral issues associated with organic diseases
Urinary disorders	Bamberger and Houpt [157] found that 28% of cats exhibiting improper elimination behavior had urinary tract infections. Also, the pain and suffering associated with them has the capacity to provoke aggressiveness and affect social interactions
Neurological conditions	Conditions like feline hyperesthesia syndrome, characterized by frenetic behavior and skin twitching, can have a significant impact on cat behavior. Other neurological problems like seizures and cognitive dysfunction syndrome (CDS) in senior cats might manifest as behavioral abnormalities [149] Deterioration in sensory functions such as hearing and vision, particularly in older cats, can result in increased vocalization, confusion, and fear-related behaviors [151]. Also, sensory deterioration might reduce spatial awareness, resulting in anxiety [149, 151]
Dental and oral disorders	Pain stemming from dental issues, often overlooked, can result in a reluctance to eat, pawing at the mouth, and aggressive behavior. According to Bellows et al. [150], dental disorders affect approximately 70% of cats over the age of five, changing appetite and behavior
Endocrine disorders	Hormonal abnormalities, like hyperthyroidism, can cause aggressive, hyperactive, and erratic behavior. Approximately 10% of senior cats developed hyperthyroidism, showing significant behavioral changes [152, 153]
Gastrointestinal disorders	Inflammatory bowel disease or gastrointestinal parasites can cause discomfort, resulting in aversion to the litter box and changes in food intake. Guilford [154] addresses how gastrointestinal disorders might influence behavior by causing discomfort and limiting food absorption

## Data Availability

No new data were generated or analyzed in this study. Data sharing is not applicable to this article.
